# Planning and conducting cross-cultural qualitative research: a methodological framework and resources for health researchers

**DOI:** 10.1080/17482631.2025.2556350

**Published:** 2025-10-01

**Authors:** Cassie E. McDonald, Catherine L. Granger, Louisa J. Remedios

**Affiliations:** a Department of Physiotherapy, The University of Melbourne, Carlton, Australia; b Department of Allied Health - Physiotherapy, The Royal Melbourne Hospital, Parkville, Australia; c Allied Health, Alfred Health, Melbourne, Australia; d Department of Physiotherapy, Federation University, Churchill, Australia

**Keywords:** Cross-cultural, qualitative, framework, ethical, methodological, healthcare, culturally and linguistically diverse

## Abstract

**Purpose:**

The aim of this review was to develop a methodological framework that could be applied during the design and conduct of cross-cultural qualitative research with people from culturally and linguistically diverse backgrounds in health contexts or settings.

**Methods:**

Developing the methodological framework in this study consisted of three phases. In Phase 1, a literature review was undertaken to identify relevant evidence by searching bibliographic databases, online sources and relevant journals. In Phase 2, methodological and ethical concepts, issues and considerations were summarized, synthesized and consolidated into a framework. In Phase 3, the methodological framework was refined by applying it to a cross-cultural qualitative study.

**Results:**

The resulting methodological framework proposes two stages (preparation and action) and eleven key steps for designing, conducting and reporting cross-cultural qualitative research. Other practical resources (i.e. glossary of terms and detailed prompt tool) for health researchers that can be used with the framework are also presented.

**Conclusions:**

This article presents a methodological framework that can guide high-quality cross-cultural qualitative research and is intended for use by health researchers, especially those new to cross-cultural qualitative research. It has the potential to improve the inclusivity and cultural responsiveness of qualitative research.

## Introduction

Globalization and migration have contributed to increasing cultural and linguistic diversity in many populations globally. To best serve multicultural communities, healthcare services must consider contextual factors experienced by and unique to sub-groups within their community, which may be influencing their health outcomes. Problematically, people from diverse cultural or linguistic backgrounds remain underrepresented in health research (K. Murray et al., 2019). This is despite many health services providing care for diverse communities (K. Murray et al., 2019), repeated efforts to highlight this issue (Flores et al., 2021; Knepper & McLeod, 2018; O’Halloran et al., 2019; Smith et al., 2018), and ongoing efforts to identify approaches to improve participation in research (Brijnath et al., 2024; Hughson et al., 2016; Watson et al., 2025). To address this, culturally, linguistically, and ethnically diverse communities must be included in all manner of research. Where there are gaps in the understanding of the experiences, perceptions and needs of these communities, cross-cultural qualitative research has an important role to play.

Designing and conducting cross-cultural qualitative research requires that health researchers develop: an understanding of key concepts and their application; an awareness of their ethical responsibilities in this context; the ability to consider unique methodological factors and then employ safe/suitable research methods; and the capacity to reflect on their own biases/subjectivities and how these influence the research process. In our experience, developing these skills and abilities can be challenging, especially for health researchers new to cross-cultural qualitative research. To address this challenge and provide a foundation for our own cross-cultural qualitative inquiry, our team searched for an existing framework, guide or tool. Whilst we identified useful, albeit disparate, resources (described later in the section, “**Considerations in cross-cultural qualitative research”**) we were unable to locate one comprehensive tool to inform our study. To address this gap, we reviewed the literature and developed a methodological framework to guide our research design and conduct.

Methodological frameworks can provide a supporting structure for inquiry through a set of assumptions, concepts, values and/or practices (Partelow, 2023). They are tools that can guide empirical observations and assist researchers to analyse complex, non-causal yet interdependent constructs (Partelow, 2023). The benefits of methodological frameworks include enhanced quality of research leading to improved rigour and reliability of findings (McMeekin et al., 2020). By providing common concepts and connecting ideas, frameworks can aid cooperation and communication between researchers, and may even stimulate interdisciplinary engagement in an area of inquiry, enabling knowledge to be aggregated to progress a scientific field (Partelow, 2023). Methodological frameworks may be preferable to other approaches such as methodological “checklists” in qualitative research. Checklists have been criticized in qualitative inquiry for lacking fit with the broad spectrum of qualitative methods, and potentially, undermining the quality of study processes and findings (Morse, 2021).

## Defining cross-cultural qualitative research

Broadly, qualitative research encompasses a range of methods and methodologies which typically aim to explore participants’ experiences, perceptions and/or insights into their behaviour and answer research questions relating to *how* and *why* phenomena occur (Busetto et al., 2020). At the core of *cross-cultural* qualitative research is the concept of **culture**. In brief, culture refers to the customary beliefs, values, knowledge, traditions, and/or shared meanings held and transmitted between interconnected individuals (Hong & Cheon, 2017). This article focuses primarily on culture shared within social groups connected by their ethnicity, language(s), country-of-origin and/or religion; although in its broader sense, culture can pertain to groups connected through other social phenomena. The term **cross-cultural** pertains to research involving participants who identify as being from a culturally and linguistically diverse background and/or in circumstances where the researchers’ cultural and linguistic background may be different to that of the research participants (Liamputtong, 2019). The term **culturally and linguistically diverse (CALD)** is commonly used in Australia to represent the “non-Indigenous cultural and linguistic groups who identify as having cultural or linguistic connections with their place of birth, ancestry or ethnic origin, religion, preferred language or language spoken at home” (pg. 3, NSW Health, 2019). Alternative terms used to represent sub-groups within CALD populations include “migrants”, “asylum seekers”, “refugees”, and “ethnic minority”. Examples of cross-cultural qualitative research in healthcare contexts may include studies involving: participants from cultural and language backgrounds different to that of the researchers; CALD groups or communities with a particular health condition or need; or CALD groups or communities accessing (or not) particular health services; or CALD groups or communities engaging (or not) with particular health interventions. In other contexts, the notion of “cross-cultural” may also refer to research involving Indigenous communities (Liamputtong, 2010a). Cross-cultural research approaches may be particularly pertinent if: i) the majority of evidence informing healthcare and service delivery focuses on the needs of the “dominant” or “majority” group within a community; ii) disparities in health outcomes have been identified within sub-groups of the community or population; and/or iii) the experiences of particular CALD groups within the community need to be elucidated to inform healthcare design and delivery. Cross-cultural research can pertain to both quantitative and qualitative methods. This article focuses on cross-cultural qualitative research.

## Poorer health outcomes and underrepresentation in research

Cycles of health inequity, marginalization and poorer health outcomes continue to disproportionately affect CALD communities (Garad, 2017; Georgeou et al., 2023). People from CALD backgrounds experience higher burden of diseases and illnesses (Khatri & Assefa, 2022), higher mortality rates (Australian Bureau of Statistics [ABS], 2022), higher risk of mental illness (Blackmore et al., 2020; J. Chen et al., 2019), increases in medical errors (Johnstone & Kanitsaki, 2006), longer lengths of hospital stays (Divi et al., 2007) and higher rates of hospital readmissions (Lindholm et al., 2012). A systematic review by Khatri and Assefa (2022) found that people from culturally and linguistically diverse (CALD) backgrounds experience challenges at multiple socioecological levels to access and participate in healthcare. To address these systematic issues, they recommended that strategies targeting individual, community and system/policy levels are required (Khatri & Assefa, 2022). Such strategies must be tailored, culturally appropriate and evidence-informed (Khatri & Assefa, 2022).

Health research generated without representation of people from CALD backgrounds cannot be assumed to represent or meet their health needs or that the research findings are generalizable to multicultural populations (K. Murray et al., 2019). Underrepresentation of people from CALD backgrounds in health research is influenced by factors such as researcher discretion, a lack of focus on CALD populations and by disparities in research funding. Excluding people from CALD backgrounds from health research commonly occurs by “default”: without rationale for this decision or a description of its implications for the research findings (S. Murray & Buller, 2007). For example, the eligibility criteria state that only participants who speak a majority language will be included without further elaboration or justification. Alternatively, the exclusion of people from CALD backgrounds may be more explicit. For example, when the eligibility criteria state that people who speak languages other than the majority language will be excluded and this is justified by researchers due to funding constraints prohibiting the use of professional interpreters. Indeed, a fifth of clinical trials (71 out of 342 registered trials) to be conducted in Australia in 2015 excluded participants with low English language proficiency despite the high prevalence of Australians who speak languages other than English (Stanaway et al., 2017). Finally, a lack of health funding, which promotes a focus on migrant or multicultural health issues, can drive underrepresentation. Up until 2016, major research funding schemes in Australia—a very multicultural country with migrants constituting 31.5% of the population—allocated relatively small portions of the total available health funding to migrant-focused research which contributed to a low uptake of research in this area (Australian Bureau of Statistics [ABS], 2025; Renzaho et al., 2015).

## Need for inclusive cross-cultural health research

Increasing the uptake of cross-cultural approaches in clinical and health service research would facilitate increased representation of cultural, ethnic and language diversity in mainstream health research. However, increasing the volume of inclusive research is only one part of the solution. Cross-cultural research must also be culturally sensitive and carefully address unique ethical issues to ensure that the research is: first, not harmful to participants; and second, capable of producing meaningful, and actionable results which benefit participants (Liamputtong, 2008; Woodland et al., 2021). Cultural sensitivity in research refers to consideration of the historical context, cultural experiences, norms, values, beliefs, and behaviours of a distinct ethnic or cultural group in the design and conduct of a study (Resnicow et al., 1999). Previous cross-cultural studies have demonstrated nuanced differences in health experiences and thus reinforced the importance of including diverse voices in research (Fu et al., 2003; Xiao et al., 2017).

The context in which people/communities live and engage in healthcare is also relevant to cross-cultural qualitative health research. Context extends beyond culture and language. Broesch et al. (2020) argue that in social sciences, cross-cultural research should recognize historical, political, sociological and cultural forces acting on the communities and individuals of focus. Applying this approach would benefit health research too. Further, contextual factors known to affect the health outcomes of people from CALD backgrounds can include structural barriers (i.e., lack of accessible health services, lack of language services, transportation issues, or economic constraints) and socio-cultural barriers (i.e., fear of stigma, models of care that are incongruent with beliefs/perceived needs, or a lack of trust in healthcare providers) (Ayers et al., 2018; Kiselev et al., 2020). Thus, factors shaping the context in which a particular health phenomenon occurs for people who identify as CALD may include cultural, structural, sociological, economic, historical, and political factors.

Research inclusive of people from CALD backgrounds could: 1) improve individual and population-level health outcomes, and 2) inform culturally competent healthcare systems that “acknowledge the importance of culture, incorporate the assessment of cross-cultural relations, recognise the potential impact of cultural differences, expand cultural knowledge, and adapt services to meet culturally unique needs” (pg 1., American Hospital Association, 2025).

## Complexities of cross-cultural, cross-language qualitative research

Cross-cultural, cross-language qualitative research presents a unique set of challenges for health researchers. First, qualitative research is inherently language-based and therefore communication issues may be heightened (Pelzang & Hutchinson, 2017). For example, if the research team and participants have different preferred languages, communicating in a nuanced manner may be limited. Secondly, our cultural backgrounds, identities and experiences shape our view of reality (Wilson, 2019). If the researcher(s) and participant(s) do not share similar cultural backgrounds, important cultural meanings and contexts may remain hidden, be misunderstood, or misrepresented by researchers (Temple, 2002). Thirdly, cross-cultural qualitative research raises unique methodological considerations that must be carefully considered and addressed by researchers when designing and reporting such studies (Hennink, 2008). These challenges may be overcome with sensitive, self-aware, and reflective research design and planning.

## Considerations in cross-cultural qualitative research

To the knowledge of the authors there are no recently published introductory toolkits or guides for health researchers new to cross-cultural qualitative research. However, there are numerous extensive and excellent resources published in a range of mediums (i.e., journal articles, textbooks) which drill down into particular methodological issues e.g (Awad et al., 2016; Burnette et al., 2014; Hennink, 2008; Squires, 2009). For health researchers new to cross-cultural research, it can be overwhelming to synthesize these issues and incorporate them comprehensively into the design of the study.

Three key guidelines/resources were identified that offer nuanced and insightful guidance or reflections; however, none offered a comprehensive methodological framework. Guidelines produced by Arriaza et al. (2015) provide a useful and in-depth discussion on four methodological areas (use of interpreters/translators, language translation, data analysis, and decisions about community relationships). However, they lack guidance on other pertinent considerations. Another useful resource is the detailed and informative summary of considerations by Broesch et al. (2020) drawn from their cross-cultural research experience in the social sciences. Their article appears to presume some prior knowledge (regarding terminology and key concepts) and only focuses on three aspects of cross-cultural research: study site selection, community involvement, and research design and methods. Pelzang et al (2017) describe in-depth their unique approach to maintaining cultural integrity in a study set in Bhutan. They present thoughtful and nuanced reflections on how their approach was framed around Bhutanese cultural values. Their sophisticated approach was developed when the lead researcher who identified as sharing the “same racial, ethnic and cultural background” as participants undertook health research in Bhutan (pg. 5, Pelzang et al., 2018). Therefore, it may not be easily adopted or adapted for use in contexts where the researcher: i) is an “outsider” to participant culture/language or ii) conceptualizes health in a manner that differs from participants due to language and culture or iii) has experiences of health systems or traditions that differ from those of participants based on language and culture. With these strengths and limitations in mind, our article adds to the extant literature by offering researchers working in clinical or health service settings a comprehensive framework to guide the nuanced, ethical, and methodological design and conduct of cross-cultural qualitative research. It also introduces key terms and concepts. As an overarching framework, this article does not exhaustively cover the minutiae of design details for all cross-cultural qualitative study designs across all contexts or settings. Rather, it presents a flexible set of tangible actions that can be applied by researchers in their setting/context. The framework consists of a “preparation stage” followed by an “action phase”. Within each phase are key steps for consideration. Although the steps are numbered for clarity in this article, the framework is designed to be used flexibly: for example, researchers may apply components of the framework iteratively, or in a sequence that best suits their study design, or omit steps that do not apply in their setting/context. Certain steps are likely to transcend both the preparation and action phases, such as, considering researcher positionality and reflexivity. Ultimately, the purpose of the framework is to prompt careful methodological and ethical consideration by researchers undertaking cross-cultural qualitative research in health contexts.

## Aims

The aim of this article is to present a methodological framework that can be applied during the design and conduct of cross-cultural qualitative research in health contexts or settings.

## Materials and methods

Developing the methodological framework consisted of three phases informed by McMeekin et al. (2020): Phase 1 – identifying evidence to inform the methodological framework; Phase 2 – developing the methodological framework; and Phase 3 – evaluating and refining the methodological framework.

### Identifying evidence via a literature review and developing the methodological framework

To develop the methodological framework, the lead author first conducted a literature review (last search in February 2022) (McMeekin et al., 2020). The search strategy included searching bibliographic databases Ovid Medline and Ovid Embase; an online search engine Google Scholar; and a targeted search of international peer reviewed qualitative research focussed journals (such as The Qualitative Report, Qualitative Health Research, International Journal of Qualitative Methods, Qualitative Research, Qualitative Inquiry, and International Journal of Qualitative Studies on Health and Well-being). Search terms included: cross-cultural, culture, cultural, language, bicultural, bilingual, culturally and linguistically diverse, migrant, qualitative, research methods, methodology, health services, health sciences, social sciences. All sources that described ethical and/or methodological considerations for undertaking cross-cultural qualitative research in social or health settings were downloaded in full text or retrieved from libraries in hard-copy (e.g., books not available online) and reviewed. Reference lists of relevant sources were hand-searched and relevant sources retrieved. Sources identified through the authors’ networks were also retrieved.

Next, key ethical and methodological concepts or steps identified in included sources were analysed by one researcher (CM) using a general inductive approach (Thomas, 2006). Initially, categories were developed from the data (i.e., ethical issues, research considerations and methodological processes). An iterative process of reviewing literature and adding to the developing categories ensued. Then, these categories were abstracted into “steps” meant to represent the research process and organized in chronological order from study inception to completion (where possible). Overlapping and/or similar concepts or steps were synthesized and consolidated. Developing categories and themes were discussed and debated during regular meetings with senior researcher (LR) throughout this process which helped to refine the key steps articulated in the framework and how the methodological considerations were represented in each stage. Broader discussions amongst the author group further helped to refine the developing framework, particularly when it was applied to a cross-cultural qualitative study led by our team. Sources that informed the framework were catalogued in Endnote reference management software (Clarivate, 2025).

As a result of this process, eleven key steps were developed and incorporated into a methodological framework. Concurrently, frequently used terms and phrases across sources were recorded and a glossary with contemporary definitions was developed. These terms and phrases are highlighted in **bold** throughout this article and the complete glossary is available in Supplementary File 1. A detailed resource with prompts for health researchers was also developed: the CONSIDER CROSS-CULTURAL Tool (see Supplementary File 3). This tool is designed to support engagement with and application of the framework during study design and conduct.

### Evaluating and refining the methodological framework

In parallel to the development of the framework, the authors were designing and conducting a cross-cultural qualitative study in a hospital outpatient rehabilitation setting which is published elsewhere (McDonald et al., 2024). The developing methodological framework was applied during the design and conduct of this study. Gaps, issues, limitations and strengths of the developing framework were identified as it was piloted. The experiences and reflections recorded by the research team in memos throughout the qualitative study enabled refining of the methodological framework.

### Research team and positionality

The research team involved in developing this methodological framework consisted of three clinician-researchers. The lead researcher, CM, is an Australian-born woman with British and Scottish immigrant heritage. At the time of this review, she was a PhD candidate with over 10 years of clinical experience working as a physiotherapist in a public health service that served a multicultural community. Through her professional experience working with patients and their families who identified as CALD in healthcare settings, she felt committed to improving the cultural competence of her own clinical practice. As she was new to cross-cultural qualitative research, she was motivated to undertake this review to improve her understanding of the nuances and complexities of cross-cultural qualitative research, to then apply these learnings to her own cross-cultural qualitative study.

The second author, CG, is an Australian-born experienced academic and physiotherapy clinician-scientist. She has extensive experience leading health services research in a large, tertiary hospital in metropolitan Melbourne which serves a multicultural community. At the time of this review, she had limited experience with cross-cultural qualitative research methods specifically. However, she was interested in exploring how such methods could be feasibly applied in healthcare settings to inform improvements in inclusive research practices in the department within which she held a senior research-capability-building position.

The senior author, LR, is an experienced academic with prior experience leading cross-cultural qualitative research. As an immigrant, she identifies as culturally diverse, although she is a fluent English language speaker (the national language in Australia). She actively promoted and encouraged co-researchers to consider the benefits of inclusive practices and the potential harms of exclusive research. She regularly advocated for CM and other early career researchers to improve their awareness of culture and its implications for research participation, design and the generalizability of findings. She strongly encouraged CM to engage deeply and critically with the literature on cross-cultural qualitative research during her PhD studies which sparked the idea for this review.

## Results: methodological framework and proposed key steps

**Figure 1.**
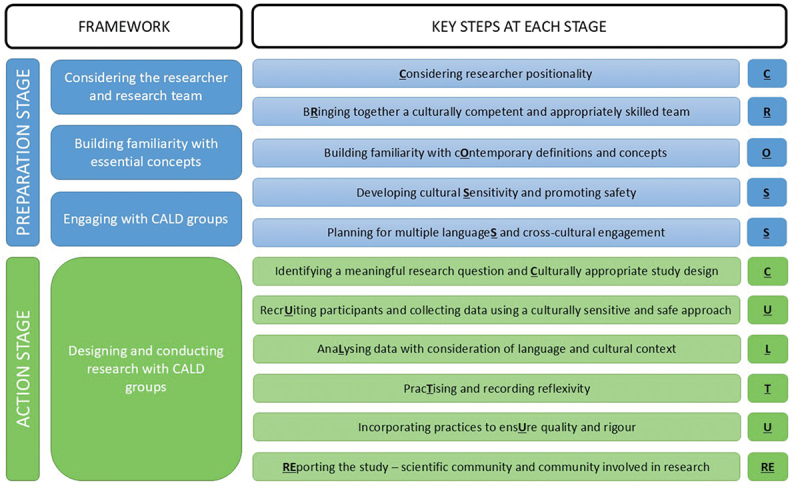
CROSS CULTURE methodological framework for cross-cultural qualitative research and proposed eleven steps.

The CROSS CULTURE methodological framework has two stages: preparation and action (Figure 1). The preparation stage includes: considering the researcher and research team; building familiarity with essential concepts; and engaging with CALD groups. The action stage involves the study design (e.g., writing a protocol) and the actual conduct of the study. The key steps proposed for each stage are outlined in Figure 1 and described in detail below. The CROSS CULTURE Framework is designed to be used in a fluid and iterative manner; the proposed key steps are unlikely to be completed in a linear or successive manner. For example, the step pertaining to “considering researcher positionality” will commence during the preparation stage and continue throughout the action stage.

### Preparation stage

There are a range of decisions and preparatory steps to consider when preparing for **cross-cultural** qualitative research. Five key steps are proposed to begin during the preparation stage.

#### Step 1: Considering researcher positionality

An early step in preparing for cross-cultural research is for the researcher to consider their positionality, sometimes referred to as subjectivity (Arriaza et al., 2015). A researcher’s positionality will affect how a study is designed and conducted because cultural and personal identity “permeates all we do” (Brion & Rogers-Shaw, 2022, p. 2094). Considering one’s own positionality involves examining with criticality one’s own subjective experiences, cultural views and belief systems in the context of the research (Johnson, 2020). Rankl et al. (2021) suggest researchers examine factors such as their gender, socioeconomic status, ethnicity, occupation, interests, assumptions and life experiences (Rankl et al., 2021). Building self-awareness through this process is proposed as a “significant step to identifying [potential] collaborators” for the research team (Arriaza et al., 2015, p. 85). Self-reflection may assist researchers to identify their motivation for engaging in a study, selecting a study population and personal impressions or assumptions about the phenomenon of interest (Arriaza et al., 2015). Considering researcher positionality is the beginning of the reflexive process which will continue throughout the course of the study (*see Step 9*).

#### Step 2: Bringing together a culturally competent and appropriately skilled research team

Building a research team involves establishing the mix of skills, experience and resources necessary to undertake the research. A culturally competent research team has the capability to identify and manage language, cultural and other barriers which may affect participation, interpretation, dissemination or implementation of findings into practice (Woodland et al., 2021). The personal attributes and qualities of cross-cultural research team members should also be considered.

If a study is not being led by researchers who share similar cultural, ethnic and linguistic backgrounds with prospective participants, finding and integrating such researchers within the team can provide insights into the cultural context of the individuals/communities who the researchers wish to learn from; this ideally occurs during study conceptualization (Liamputtong, 2010a). When enacted in the formative stages of a study, and embraced by the research team, such insights into the cultural context of individuals/communities can transmute the premise of a study and its research question(s). Working collaboratively from the outset with researchers who can serve as cultural/contextual brokers (explored further in Step 4) can also foster cultural sensitivity and competence amongst the team, which in turn can positively shape design choices and research conduct (L. Chen et al., 2021; Liamputtong, 2010b).

#### Step 3: Building familiarity with contemporary definitions and concepts

Researchers must first familiarize themselves with contemporary definitions of **culture** (Kwantes & Glazer, 2017; Wilson, 2019). They should then consider the following general principles (defined in Table I) to uphold the **cultural integrity** of a study: cultural relevance, contextuality, appropriateness, mutual respect and flexibility (Pelzang & Hutchinson, 2017). The concept of **intersectionality** may also be relevant to some cross-cultural qualitative studies (revisited in Step 6).

**Table I.** General principles for upholding the cultural integrity of cross-cultural studies adapted from Pelzang and Hutchinson^a.^

General principle	Brief description
Cultural relevance	The study research question and aims will benefit and improve the lives of the target population and is informed by cultural knowledge and context.
Contextuality	Sensitivity and awareness are given to contextual factors which shape participant’s responses. Contextual factors may include cultural values, previous experiences, socio-political pressures or other ethical considerations.
Appropriateness	Appropriate language and communication styles are used in the study. Appropriate translation processes have been devised to accurately convey concepts and meanings shared by participants.
Mutual respect	The cultures of the participants and researchers are mutually respected. This includes upholding respect for the values and beliefs of research participants and that every effort has been made to minimize any risk to participants (i.e., privacy and confidentiality).
Flexibility	Researcher approaches the study with a flexible approach to ensure that study participants feel comfortable and able to exercise their choices. Examples of this may include: Offering participants the choice to conduct their interview in their preferred language or ensuring that participants feel comfortable to decline or withdraw from the study.

^a^Pelzang and Hutchinson (2017).

#### Step 4: Developing cultural sensitivity and promoting safety

Consideration of **cultural sensitivity** and safety commences at the inception of the research question and continues through to dissemination of the research findings. To develop cultural sensitivity, researchers must acquire “cultural knowledge of the social group that [they] wish to learn from” (Liamputtong, 2008, p. 4). Learning about aspects of the community of interest may include their history, customs, interactions and communication habits (Colucci, 2008). Researchers can demonstrate cultural sensitivity by: 1) developing an understanding of the cultural context, diversity within and key values of the cultural group; 2) adopting culturally appropriate communication approaches; and 3) exhibiting a willingness to learn (Liamputtong, 2008). Admittedly, these important collaborative practices can be complex, costly, and time-consuming to implement. Explicitly planning for and factoring these practices into the research design, estimated timeframes and the project budget will aid their incorporation throughout.

**Culturally safe** research adheres to the principles of partnership, participation, and protection to ensure that participants feel included, respected, and able to trust the integrity of the research (Wilson, 2019; Wilson & Neville, 2009). Culturally safe research should reflect the voices and needs of the participants, and the findings should be relevant, useful, and beneficial to the target population (Wilson & Neville, 2009). Cultural safety requires researchers to identify their own values, beliefs, and practices and then consider how these may influence research involving participants with different cultural realities (Wilson & Neville, 2009).

#### Step 5: Planning for multiple languages and cross-cultural engagement

If the research team do not share the same cultural and language background as the research participants, collaborating with one or more **language assistants** and **cultural brokers** is crucial. Allowing participants the opportunity to participate in research in their preferred language is essential to ensuring that the participant can express what they want to say and therefore to optimize the quality of data (Choi et al., 2012). As well as overcoming the language barrier, language assistants and/or cultural brokers provide essential cultural insights. Without cultural knowledge, there is a risk that participants’ data may be misinterpreted or misrepresented (Arriaza et al., 2015). The two most common language assistants employed in cross-cultural data collection are **formal language interpreters** or **bilingual researchers**. The nuance of this debated decision is explored in-depth elsewhere e.g (S. K. Lee et al., 2014; Temple, 2002);. a brief summary is provided in Supplementary File 2 to illuminate this issue. Temple argued back in 2002 that the bilingual researcher model was preferred as it acknowledges their active participation in the research proceeds as cultural and linguistic information is exchanged, interpreted, and analysed (Temple, 2002). Likewise, several research teams have advocated for including researchers who can serve as cultural brokers in the research team and espoused the advantages for the quality and impact of their research (Arriaza et al., 2015; Blackman et al., 2024; L. Chen et al., 2021; Pelzang & Hutchinson, 2017; Stapleton et al., 2014).

Sometimes, the choice between an interpreter or bilingual researcher model may be influenced by practical considerations such as availability and cost. If a language assistant is engaged in the research team, their role should be clearly defined and training in cross-cultural qualitative research offered. Ensuring clear understandings of roles and training of language assistants and cultural brokers will increase sensitivity to the research topic and aims (Hennink, 2008).

**Figure 2.**
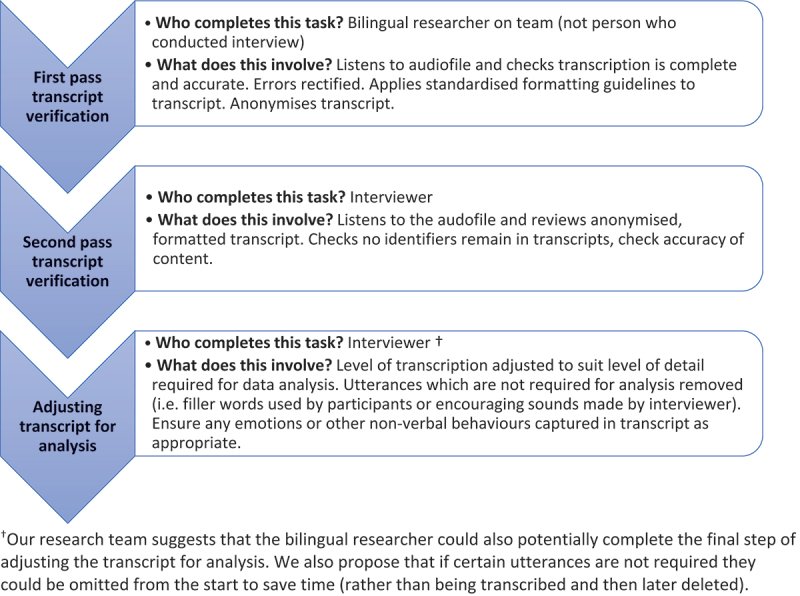
Summary of the multi-step process for verifying translated interview transcripts based on Clark et al. (2017).

^†^Our research team suggests that the bilingual researcher could also potentially complete the final step of adjusting the transcript for analysis. We also propose that if certain utterances are not required they could be omitted from the start to save time (rather than being transcribed and then later deleted).

Data collected in multiple languages or where the language(s) differ from the primary language of the local region or country may require **verbal interpretation** or **written translation** to be shared with a broader audience. This role of transcribing and translating participants’ spoken words into written text involves interpreting their meaning and deciding how to represent this meaning in the language of the research team (Clark et al., 2017). Concept validity, in the context of translation, is defined as achieving **conceptual equivalence** between two languages and cultures (Choi et al., 2012). Cross-cultural researchers have raised concerns about the validity of data translations when poorly defined translation procedures are used or only a vague account of these procedures is reported (Squires, 2009). Poorly translated data may not accurately reflect what participants said, and this may change the themes developed in analysis (Squires, 2009). To improve the accuracy of cross-cultural data translation, the following steps have been proposed: involve two or more translators; undertake cycles of translation and **back-translation**; conduct debrief interviews with language assistants after research interviews and document each translation decision in an audit trail (Clark et al., 2017). Additionally, Clark et al. (2017) proposed a multi-step process for verifying translations, which is summarized in Figure 2. The timing of translations should be justified (Santos et al., 2015) with five potential timepoints to consider: 1) prior to data collection; 2) at data collection; 3) during data preparation; 4) during data analysis; or 5) at dissemination of findings (Santos et al., 2015).

At times, pragmatic decisions may be necessary when designing the translation process, depending on constraints such as funding and time. For example, independent back translation of transcripts can be costly and may not be feasible. Researchers need to be aware of these costs and could include them in research funding applications (Irvine et al., 2008). These pragmatic decisions should be transparently reported and explained when disseminating findings.

### Action stage

There are six key steps proposed within the action stage. These six steps highlight the unique considerations and actions required when designing and conducting qualitative studies in cross-cultural contexts.

#### Step 6: Identifying a meaningful research question and appropriate study design

To develop the research question and design the study, identifying what is important and meaningful to the individuals and community of focus is critical. Unethical “extractive” approaches whereby the researcher nominates a community of interest and collects necessary data, without clear benefits for the participating community, must be avoided (Broesch et al., 2020). Broesch et al. (2020) recommend that the step of community engagement/participation involve a two-way dialogue between researchers and prospective participants. Increasingly, consumers with lived experience are engaged in research teams or on project steering committees to contribute to this agenda. Arguably, there is no “perfect solution” for community engagement; this step warrants careful consideration of power, representation, and potential burden for participants.

As the study is conceptualized, the notion of intersectionality could be considered. Particularly, whether applying this theoretical lens may be relevant to the research question, target population, and the health outcome of interest. Exploring participants’ experiences at the intersection of multiple identities/characteristics (i.e., gender, race, religion) may elicit an understanding of unique experiences that may be missed by focusing on a singular identity (Kelly et al., 2022). Intersectionality in the context of health research “foregrounds” the marginalized voices of people who can shed light on the structures of oppression that may be influencing their health outcomes (Kelly et al., 2022) (See Box 1 for an example).

**Box 1 Example of the concept of intersectionality used to inform a cross-cultural qualitative study**

In their qualitative intersectionality study, Brady et al. (2017) explore the body image experiences of Asian American women at the intersections of gender and race. The authors reflected that examining their participants’ lived realities through multiple dimensions of their identity elicited nuanced findings into the structures of inequality that influence Asian American women’s body image and related health outcomes (Brady et al., 2017).

Explicating the study paradigm (ontology and epistemology) is also important during the design phase. Liamputtong (2010a) contends that cross-cultural qualitative research should not be rigid and therefore cannot be objectivist or positivist in its orientation: she advocates that a study aiming to “measure” or “generalise” in qualitative inquiry would risk dismissing or marginalizing the voices of participants (Liamputtong, 2010a). Instead, a flexible and interpretive approach to qualitative inquiry is recommended as better able to understand the views, experiences and perspectives of participants (Liamputtong, 2010a). Equally, selecting a suitably aligned research method/methodology is important. The selected method/methodology should be compatible with culturally appropriate modes of data collection and data analysis approaches (See Box 2 for an example of these principles in action).

**Box 2 Example of careful selection and justification of the study paradigm and research methods employed in a cross-cultural qualitative study**

Blackman et al. (2024) clearly describe the context for their study exploring doulas’ experiences assisting Black birthing families during the pandemic. The authors clearly link and justify all aspects of their study design including their epistemological approach (Black Feminist Thought), use of a relevant theoretical framework (epistemic justice), data collection methods (virtual story circles), and data analysis approach (the “Thinking with Theory” conceptual framework) (Blackman et al., 2024). Where their methods deviate from “more commonly used” qualitative methods (i.e., collecting data using virtual story circles instead of standard focus groups), they rationalize the contextual factors that shaped their design choice (Blackman et al., 2024).

#### Step 7: Recruiting participants and collecting data using a culturally sensitive and safe approach

Once the research question is defined, strategies for participant recruitment and data collection can be designed in a study protocol and actioned once ethical approval is obtained. Barriers to participant recruitment and data collection should be anticipated and strategies devised to manage these. If the research population has been subjected to marginalization or exploitation, additional obstacles may be apparent (Burnette et al., 2014). One strategy to build trust and rapport with participants is getting to know each other (i.e., meeting a few times with participants) before commencing data collection (Liamputtong, 2007); however, the potential burden on participants should be considered if this increases the time commitment for research participation. Practical and methodological considerations for data collection will vary depending on the methods/methodologies, data type, and the proposed mode of engaging with participants. For example, collecting data via online cross-language interviewing will have different inherent opportunities, challenges and considerations compared to cross-cultural virtual workshops (Chiumento et al., 2018; Fakhari et al., 2023).

Thoughtful and respectful access to individuals or communities also warrants consideration. In their critical ethnography with indigenous communities, Burnette et al. (2014) contend that relationships are integral to “gaining access” as well as incorporating Indigenous traditions and strengths in research methodology. They propose strategies for researchers such as spending time with a community, listening, working with a cultural insider, exhibiting cultural humility, exhibiting transparency and working collaboratively (Burnette et al., 2014). As these strategies emphasize cultural sensitivity and respect, they could be extrapolated to cross-cultural research with CALD populations.

Multifaceted processes may be necessary for obtaining informed consent in cross-cultural studies, to account for diverse preferred languages and varying literacy levels across languages. Strategies could include translating written participant information as well translating and culturally adapting audio-visual resources explaining the study.

#### Step 8: Analysing data with consideration of language and cultural context

When analysing data, Irvine et al. (2008) advocate for the research team to reflect on each researcher’s position as cultural “insiders versus outsiders” and how this may influence the interpretative process. Researchers who are **cultural insiders** are able to directly interpret the original data as well as the translated transcripts, which allows them to mediate and interpret both data sets. In contrast, researchers who are **cultural outsiders** may lack sensitivity to the nuance in meaning and language used by participants during analysis. Other researchers propose that this dichotomized notion of the researcher’s position (as either insider or outsider) is problematic (Pelzang & Hutchinson, 2017). Instead, they advocate for a fluid conceptualization (along a spectrum between insider and outsider) of the researcher’s identity and position at each stage of cross-cultural studies, including during data analysis (Pelzang & Hutchinson, 2017).

#### Step 9: Practicing and recording reflexivity

An essential tool for enhancing the rigour of qualitative research is reflexivity. Reflexivity is defined as the researcher’s examination of their position (*see step 1*) and how this shapes their engagement with the research process (Rankl et al., 2021). As described by Rae and Green (2016), qualitative researchers can draw from a range of approaches to reflexivity such as: bracketing—making efforts to suspend biases, epistemological reflexivity—reflecting on assumptions throughout the course of research, critical theory perspective—examining political and social conditions linked to the research, or feminist theory perspective—emphasizing power differentials in research. Reflexivity can be practised through individual reflections or group reflections (Rankl et al., 2021) and documented through writing (i.e., memos) or recording (i.e., audio or video). Reflexivity should be practised throughout a study from the inception of the research question through to completion of the research report (Arriaza et al., 2015). All members of the research team, including any language assistants, should participate in reflexivity (Hennink, 2008). Cross-cultural researchers argue that this makes the language assistant more “visible” and aids a sense of accountability for all researchers (Hennink, 2008). To prompt language assistants to engage in reflexivity the following issues could be explored and noted: personal characteristics of assistants; their personal ideas or opinions on the research topic; experience of the topic or any preconceived ideas or agendas; relationships with the study community; their language competency and other sociocultural factors (such as social, familial, cultural, religious, historical and political backgrounds) (Hennink, 2008). S. Lee (2017) has also described how reflexivity can overcome translation issues through transparent reporting of: i) the interpreter’s/bilingual researcher’s language biography to heighten awareness of how they may have approached or influenced the translation process; and ii) challenges experienced during translation and how these were resolved by the research team.

#### Step 10: Incorporating practices to ensure quality and rigour

Qualitative research has a different set of quality criteria from that used in quantitative research. The four criteria for maintaining rigour in cross-cultural qualitative research, originally conceptualized by Lincoln and Guba (1985) and subsequently adapted by Irvine et al. (2008), are proposed as: 1) credibility; 2) transferability; 3) dependability; and 4) confirmability. Strategies for maintaining rigour according to these four criteria are outlined in Table II.

**Table II.** Practical strategies for maintaining rigour in cross-cultural research adapted from multiple sources.

Criterion	Practical strategies for addressing criterion
Credibility^a^	Collect data in participants’ preferred language Involve research team members who share the language and culture of participants Pre-define and standardize translation procedures for all data Conduct peer debrief throughout the research process Report a detailed description of the translation methods
Transferability^b^	Report the demographic profile of participants including their language profile and the research setting
Dependability^b, c^	Involve an independent person to conduct data analysis, or compare and discuss findings between multiple analysts Conduct member checking with participants to check that the findings resonate with their experiences
Confirmability^b^	Document procedures and decisions transparently in an audit trail

^a^(Arriaza et al., 2015).

^b^(Liamputtong, 2008).

^c^(Burnette et al., 2014).

#### Step 11: Reporting the study—scientific community and community involved in the research

When reporting the research findings, cultural sensitivity and cultural safety should be ensured (Burnette et al., 2014; Wilson & Neville, 2009): the report should be respectful, nuanced, and avoid stereotypes. To achieve this, member checking with participants as well as collaborative writing with community members and/or CALD-consumer lived-experience experts on the research team and/or language assistants may be useful strategies (Arriaza et al., 2015). Burnette et al. (2014) encourage using a **cultural reader** to review materials prior to dissemination and to prevent inadvertent publication of harmful material.

Most international journals publish in the English language, which requires extra attention when translating and reporting cultural meanings from participants’ original language (Irvine et al., 2008). Reporting findings beyond traditional avenues of academic publications or conference proceedings is also important to ensure that the CALD community can access the findings (Awad et al., 2016). Therefore, the dissemination plan should be ethically informed by what is most appropriate, accessible to, and preferred by the participating community.

## Discussion

This article proposes a comprehensive methodological framework with eleven key steps that can be applied by health researchers designing and conducting research with a cross-cultural dimension: thereby addressing a gap in the literature on cross-cultural qualitative research methods. In addition to the novel framework, this article draws together and cites many excellent and detailed resources that health researchers can use to further develop their knowledge of specific elements of cross-cultural research methods. We anticipate that demand for such resources will continue to increase as many health services provide care for multicultural populations. A critical challenge for health services that provide care to communities within which multiple languages are spoken is the ethicality of representation. When considering representation in multicultural communities, it raises the question regarding which perspective(s) should be given priority or included? One approach could be to review local data on health outcomes within population sub-groups. Then, participants from sub-groups with poorer health outcomes could be prioritized for inclusion in subsequent research.

### Strengths of the methodological framework

A strength of the CROSS-CULTURE methodological framework proposed in this article is that it provides a clear and comprehensive overview of key considerations for cross-cultural qualitative research. The accompanying glossary and the CONSIDER CROSS-CULTURAL Tool are practical resources that can be applied by health service researchers and may prompt deeper engagement with the framework. Another strength is that the framework can be applied across different qualitative study designs and therefore it may be widely used.

Applying this methodological framework in a recent cross-cultural qualitative study (McDonald et al., 2024), usefully informed how the authors prepared for, designed, and conducted the study. In our experience, the framework facilitated a systematic approach to preparing our study. During the preparation stage, our team considered each component of the framework, used the prompt questions to consider the implications of each design choice and enacted the recommended steps whilst preparing our team and study materials. Then, during the action stage, the framework helped us to ensure that we implemented the key considerations in our study. The prompt questions helped us to be thorough, considered, and kept us accountable to undertaking and reporting the study in a nuanced manner. A specific example as to how the framework and supplementary tools aided our study was elucidated during the step “bring together a culturally competent and appropriately skilled team”: during this step the researchers identified that bilingual health professionals employed at the study site could be a good fit to join the study team as bilingual researchers. The CONSIDER CROSS-CULTURAL Tool prompted our team to delve into the methodological literature regarding involving health professionals as bilingual researchers (see exemplar summary of considerations in Supplementary File 4). This demonstrates how the framework and its associated resources can prompt further exploration of extant literature to inform and justify specific aspects of study design.

Applying the framework to our qualitative study also enabled critical reflection of our methodological decisions and helped us to identify areas for improvement in future studies. For example, in retrospect, further engagement with CALD communities during the research question development and study design phase was needed in our study. In this particular study, we relied heavily on input from bilingual researchers to inform the study design and adapt our study procedures. In future, we could extend this approach and incorporate broader, meaningful community participation or engagement at the project inception. Thus, another strength of the framework is that it can be used by researchers to critically reflect on and learn from their methodological choices. This may lead to enhanced study design and conduct of cross-cultural qualitative studies in health settings, although this warrants empirical testing.

### Limitations of the methodological framework

A notable limitation of this framework is that it was developed from a literature review. As this was not a systematic or scoping review, it is possible that some concepts or methodological considerations were missed. Future research could involve completion of a systematic or scoping review to identify all sources on this topic, which potentially could further refine the framework. Although the framework is empirically informed from a review of the literature, it may be strengthened by testing or consultation with participants from CALD backgrounds.

Another potential limitation of the framework is that it has only been applied to a single cross-cultural qualitative study to date (McDonald et al., 2024). Future application of the framework to a range of cross-cultural qualitative studies as well as critical appraisal by cross-cultural research teams may assist with exploring the sufficiency of the proposed stages and steps: should additional important ethical or methodological considerations be identified, these could be incorporated into future iterations of this framework.

### Implications for research and practice

Cross-cultural qualitative research presents important benefits for the research participants and unique learning opportunities for the research team. A rewarding aspect of cross-cultural research is the opportunity for researchers to develop cultural sensitivity, competence, and self-awareness, thanks to the sharing of culture and engagement in reflexive practices. When research teams include clinician-researchers, we posit that heightened cultural sensitivity and awareness may inform and improve cross-cultural clinical practice. More importantly, for the communities of participants, cross-cultural research ensures their health experiences and needs are included in the discourse and evidence informing health care design and delivery. Including CALD perspectives in health research highlights the nuanced and diverse cultural needs that may exist within a community and thereby increases the health service’s ability to address these diverse health needs (Henderson & Kendall, 2011). Providing culturally appropriate and informed care is integral to improving health outcomes and addressing issues of inequity experienced by CALD communities.

## Conclusions

The methodological framework and eleven key steps proposed in this article offers an introductory resource for health researchers new to qualitative inquiry in cross-cultural health contexts. Applying this framework may support the integration of the many complex considerations required for safe and effective cross-cultural research with CALD communities. Health care practice and policy must be informed by evidence that reflects the care needs and experiences of people from CALD backgrounds. Including people from CALD backgrounds in cross-cultural qualitative research is essential to address issues of under-representation in health research. Researchers should consider and address key ethical and methodological issues when preparing for, designing and conducting cross-cultural qualitative research.

## Supplementary Material

SupplementaryFile4_Final.docx

SupplementaryFile3_Final.docx

SupplementaryFile2_Final.docx

SupplementaryFile1_Final.docx

## Data Availability

Data sharing is not applicable to this article as no new data were created or analysed in this study.
