# Supplementary material for: Planning and conducting cross-cultural qualitative research: a methodological framework and resources for health researchers
Source: Int J Qual Stud Health Well-being. 2025 Oct 1;20(1):2556350. doi: 10.1080/17482631.2025.2556350 (PMC12490384; doi:10.1080/17482631.2025.2556350)
Supplement: SupplementaryFile4_Final.docx [file ZQHW_A_2556350_SM9504.docx]

**Supplementary File 4**

**Summary of rationale and literature supporting health professionals as bilingual researchers**

There were multiple benefits of having registered or accredited health professionals participate in the research team as bilingual researchers. First, they receive basic research training during their university degree which gives them a foundation of research knowledge and ethics. Secondly, health professionals in many countries have a common ethical code of conduct upheld through their professional registration board which further ensures high standards of ethical conduct. Thirdly, clinician-researchers bring transferable and desirable clinical skills and attributes to the research process (Hay-Smith et al., 2016). Fourthly, health professional’s clinical knowledge or familiarity with local health service processes, systems, language and traditions can inform their understanding of the health service setting being investigated (Lee et al., 2014). Finally, including health professionals offered an opportunity to upskill clinical staff in less commonly known and taught cross-cultural qualitative research methods. These benefits of inviting clinicians to participate as clinician-researchers are accompanied by a caution from Hay-Smith et al. (2016) of potential drawbacks such as “role confusion”. Role confusion is where researchers experience tension or conflict between their clinical duty and the requirements of their role as a researcher. An example of this may be managing personal, clinical queries from participants during a research interview. Managing this dual-role as clinician-researcher can be problematic e.g., if it blurs boundaries (Hay-Smith et al., 2016). They suggest that this dual-role should be discussed when designing the study and a plan devised to manage any potential risks regarding researcher roles (Hay-Smith et al., 2016).

In the planning stages of this study, before deciding on inviting health professionals to participate as bilingual researchers, for practical reasons we considered utilising formal language interpreters. The study site serves a multicultural community. As with similar services, they use professional, accredited language interpreters to interpret clinical encounters between staff and patients who speak different languages (Karliner et al., 2007). Professional interpreters have been shown to improve clinical care and therefore serve an important role in health service provision (Karliner et al., 2007). Professional interpreters already employed by or known to the health service seemed like a convenient, accessible and cost-effective option for interpreting research interviews. However, using an interpreter (who is not trained in qualitative research) to assist during a research interview has been argued to have limitations such as: risk of missing nuances which convey meaning about the research topic (Björk Brämberg et al., 2013); and lack awareness of ethical principles required when facilitating research (Shklarov, 2007). Benefits of using bilingual researchers are their potential to be a cultural broker through their dualistic position: they may be able to perceive dual conceptual meanings and navigate cross-cultural perspectives throughout the research process (Shklarov, 2007). Based on these considerations and the research paradigm underpinning our study we decided on the bilingual researcher model.

**References (Supplementary File 4)**

Björk Brämberg, E., and Dahlberg, K. (2013) Interpreters in cross-cultural interviews: A three-way coconstruction of data. *Qualitative Health Research,* 23(2), 241-247. [https://doi.org/10.1177/1049732312467705](https://doi.org/10.1177%2F1049732312467705)

Hay-Smith, E.J., Brown, M., Anderson, L., and Treharne, G.J. (2016) Once a clinician, always a clinician: A systematic review to develop a typology of clinician-researcher dual-role experiences in health research with patient-participants. *BMC Medical Research Methodology,* 16(1), 95. <https://doi.org/10.1186/s12874-016-0203-6>

Karliner, L.S., Jacobs, E.A., Chen, A.H., and Mutha, S. (2007) Do professional interpreters improve clinical care for patients with limited English proficiency? A systematic review of the literature. Health Services Research, 42, 727-754. <https://doi.org/10.1111/j.1475-6773.2006.00629.x>

Lee, S.K., Sulaiman-Hill, C.M., and Thompson, S.C. (2014) Overcoming language barriers in community-based research with refugee and migrant populations: Options for using bilingual workers. *BMC International Health & Human Rights,* 14(1), 1-22. <https://doi.org/10.1186/1472-698X-14-11>

Shklarov S. (2007) Double vision uncertainty: The bilingual researcher and the ethics of cross-language research. *Qualitative Health Research,* 17(4), 529-538. [https://doi.org/10.1177/1049732306298263](https://doi.org/10.1177%2F1049732306298263)
