# Supplementary material for: Planning and conducting cross-cultural qualitative research: a methodological framework and resources for health researchers
Source: Int J Qual Stud Health Well-being. 2025 Oct 1;20(1):2556350. doi: 10.1080/17482631.2025.2556350 (PMC12490384; doi:10.1080/17482631.2025.2556350)
Supplement: SupplementaryFile3_Final.docx [file ZQHW_A_2556350_SM9503.docx]

**Supplementary File 3**

**CONSIDER CROSS-CULTURE Tool: Prompts for health researchers to CONSIDER when preparing, designing and conducting CROSS-CULTURE qualitative research**

*For every question or action listed below we encourage researchers to ask themselves:*

1. *“What are the implications of this choice?”*
2. *“Have I justified this choice?”*
3. *“Have I ensured that cultural sensitivity was maintained and the well-being of my research participants was the foremost consideration when making this choice?”*

| **Stage** | **Step** | **Prompt questions to consider / actions to complete^[[1]](#footnote-1)^** |
| --- | --- | --- |
| **Preparation** | 1: Considering researcher positionality | - Reflect on one’s subjective experiences, cultural views and belief systems relevant to the research topic or idea - Develop self-awareness through reflection:   - What motivates the researcher(s) to engage in this study?   - Why has the study population been selected?   - What personal impressions or assumptions about the phenomenon of interest do the researcher(s) hold?   - What skills and experience does the primary researcher lack which will be required within the skill-mix and experience of the research team? |
|  | 2: Bringing together a culturally competent and appropriately skilled research team | Does the research team:   - Have team members with evidence of cross-cultural qualitative research knowledge and skills? - Need any further training in any skills / methods i.e., qualitative interview training? - Have knowledge of the healthcare setting for the study? - Have cultural and language knowledge matched with the target population? - Do the research team members present with desired qualities ^a^: tolerance for ambiguity, patience, adaptiveness, capacity for tacit learning, and courtesy? |
|  | 3: Building familiarity with contemporary definitions and concepts | - Familiarise the research team with contemporary definitions of important terms and concepts - Consider how cultural integrity will be upheld ^b^: cultural relevance, contextuality, appropriateness, mutual respect and flexibility |
|  | 4: Developing cultural sensitivity and promoting safety | - Build relationships with and get to know the community who will be engaged in the research - Consider the history, customs, interactions, communication preferences, diversity within and key values of the community/cultural group - Adopt culturally considerate communication approaches and exhibit a willingness to learn from the community - Adhere to principles of partnership, participation and protection to ensure cultural safety throughout the study - Reflect on the values, beliefs and practices of the research team and consider how these may influence the study (see also, steps 1 and 9) - Factor in the time, resources and budget in the study plan that will be required to build collaborative relationships |
|  | 5: Planning for multiple languages and cross-cultural engagement | - Is a language assistant and/or cultural broker required? - Which model of language assistant will be used in this study (e.g., interpreter or bilingual researcher)? - Has a justification for the choice of model been provided which considers the research paradigm, methodology, and any other practical considerations or constraints? - How and when will the language assistant provide language and cultural interpretation/translations during the study? - Are there any other ethical considerations or factors which need to be planned for and managed? |
|  |  | - Create a clear and well-defined role description for the language assistant - Consider and negotiate how their role can best contribute to each stage of research |
|  |  | - Draw on available tools and information to complete training with the research team, including the language assistant - Tailor training to the knowledge and experience of the ream member. If they are new to cross-cultural qualitative research, training should include:   - an introduction to cross-cultural qualitative research ethical and methodological considerations; and   - specifics of the research study i.e., research questions, aims, methods et cetera |
|  |  | - Discuss cultural considerations for target population for the study - Develop cultural awareness amongst all members of the research team |
| **Action** | 6: Identifying a meaningful research question and culturally appropriate study design | - Consider – will this research benefit the target population and improve the lives of research participants? - Are there any safety or well-being considerations to ensure non-maleficence? - Are there any ethical or historical factors which may influence how the target population perceive research generally or this research topic specifically? - Who else should have a say or contribute to the design of this study? - Should one or more representatives from the cultural and language group join the research team or be consulted during the study design? - To uphold cultural integrity, how will the following principles inform the study design: cultural relevance, contextuality, appropriateness, mutual respect and flexibility? |
|  | 7: Recruiting participants and collecting data using a culturally sensitive and safe approach | *Recruiting participants:*   - Devise a plan for advertising the study, approaching and recruiting participants - Ensure recruitment procedures are culturally informed and sensitive - Consider how the researchers will gain the trust of the community and build rapport with participants - Ensure participants are well informed of their rights, have sufficient opportunity to ask questions or discuss the study with something they trust external to the study team and feel comfortable to decline participating in the study if they do not wish to be involved |
|  |  | *Obtaining informed consent:*   - Design a research-participant centred informed consent process - Design study materials (i.e., plain language information sheets) which are culturally informed and accessible for prospective participants - Offer for potential participants to bring along a support person to informed consent meetings - Consider how best to build rapport and trust prior to obtaining informed consent if this is likely to reduce participant suspicion and increase participation rates without resulting in unacceptable burden on the participant - Respond to participants’ questions about the study until they are clear what the study is about and what participation will entail - If appropriate, design flexible options for obtaining consent (i.e., written informed consent versus audio-recording an electronic statement) so that participants can choose the most appropriate option for them – seek relevant ethical approvals for these processes prior to commencing recruitment |
|  |  | *Collecting data:*   - What data needs to be collected to answer the research question? - Who would be best placed to collect the data and why? - What is the most appropriate way of collecting and recording this data? - How can participants be supported and reassured to feel comfortable and safe to provide honest responses? - What culturally informed communicative practices should be employed during data collection? - How can participant burden be minimised during data collection? - Will debriefs be held with the language assistants after data collection and what should be the focus of these debrief sessions? - Will field notes be recorded and what should be the focus of these field notes? |
|  | 8: Analysing data with consideration of language and cultural context | *Question before transcribing/translating data* ^c^*:*   - How will content accuracy be ensured?   *Actions before transcribing/translating data:*   - Create a clearly defined transcription/translation procedures and report these in sufficient detail to enable reproducibility of methods - When will translation occur? Consider the timing and justification for this - Decide how will decisions about translations will be documented - Will strategies be used to improve the accuracy of data translation, such as: involving two or more different translators; cycles of translation and back-translation; debriefing interviews with interpreters after research interviews and documenting in an audit trail each translation decision - What other practical considerations such as funding, timing or access to qualified translators will affect the translation protocol? - Should the research team apply for funding to support the translation process?   *Actions after transcribing/translating data:*   - Verify the accuracy of the transcribed/translated data (if this aligns with the research methodology) - Discuss the strengths and limitations of the transcription / translation procedures used in the research report |
|  |  | - Reflect on each research team member’s position along the spectrum from cultural “insider” to “outsider”. How will this person’s position impact on analytical interpretations? - How will the cultural meaning and nuance of participant’s responses be maintained during analysis? - Consider which analytical processes will be used that align with the research paradigm, study aims, researcher experience, participant cultural factors and other practical considerations |
|  | 9: Practising and recording reflexivity | - When and how will all members of the research team engage in reflexivity? - Which issues / prompts will be used to explore and note during reflexive exercises? |
|  | 10: Incorporating practices to ensure quality and rigour | - What practices are in place to generate rigorous and high quality cross-cultural qualitative research?   Specifically, what will be done to ensure:   - - Credibility   - Transferability   - Dependability   - Confirmability |
|  | 11: Reporting the study | - Check that the research report is written in respectful and nuanced language and avoids stereotypes - Write the report collaboratively with language assistant(s) / cultural broker(s) - Consider member checking the findings with participants - Have a ‘cultural reader’ review the materials prior to dissemination - Carefully consider how translating the findings for publication (if not in the original language of participants) has impacted the findings - Create a dissemination plan which enables the research participant community to access the findings |

^a^ Desired qualities as reported by Laverack, G. R., & Brown, K. M. (2003). Qualitative research in a cross-cultural context: Fijian experiences. *Qualitative Health Research, 13*(3), 333-342. [https://doi.org/10.1177/1049732302250129](https://doi.org/10.1177%2F1049732302250129)

^b^ As summarised in Table 1 and originally described by Pelzang, R., & Hutchinson, A. M. (2017). Establishing cultural integrity in qualitative research: Reflections from a cross-cultural study. *International Journal of Qualitative Methods, 17*(1). <https://doi.org/10.1177/1609406917749702>

^c^ This question was adapted from Clark, L., Birkhead, A. S., Fernandez, C., & Egger, M. J. (2017). A transcription and translation protocol for sensitive cross-cultural team research. *Qualitative Health Research, 27*(12), 1751-1764. [https://doi.org/10.1177/1049732317726761](https://doi.org/10.1177%2F1049732317726761)

1. *Many of the prompts crossover with broader considerations for qualitative research and may not be exclusive to cross-cultural research* [↑](#footnote-ref-1)
