# Supplementary material for: Planning and conducting cross-cultural qualitative research: a methodological framework and resources for health researchers
Source: Int J Qual Stud Health Well-being. 2025 Oct 1;20(1):2556350. doi: 10.1080/17482631.2025.2556350 (PMC12490384; doi:10.1080/17482631.2025.2556350)
Supplement: SupplementaryFile2_Final.docx [file ZQHW_A_2556350_SM9502.docx]

**Supplementary File 2**

**Summary of language assistant options: Interpreter versus bilingual researcher**

A formal language interpreter may be viewed as a ‘neutral’ transmitter of information between two parties. Contrastingly, bilingual researchers are perceived as actively contributing to the research process and how their own background, social context and interpretation influences the research is examined. The epistemological position assumed for a study will influence which one of these language assistant options is selected. A ‘neutral’ language interpreter, seen to be objective and separate from the research, aligns with a positivist paradigm (Temple, 2002). In contrast, a bilingual researcher is acknowledged as bringing their own personal subjectivities and sociocultural context which inherently influences the research process (Temple, 2002). Other paradigms such as social constructionism or constructivism acknowledge this influence and contend that it is a necessary part of cross-cultural research which if thoughtfully considered and managed does not detract from the rigour of the study (Temple, 2002). Such paradigms assume that the bilingual researcher contributes to the ‘knowledge-creation’ process within the research and view the transcription-translation process as “a three-way co-construction of data” between participant, language assistant and researcher (Björk Brämberg & Dahlberg, 2013; Liamputtong, 2008). Increasingly, many cross-cultural researchers are arguing that interpreting language is an act of research decision-making and therefore all language assistants actively influence the research outcomes (Björk Brämberg & Dahlberg, 2013). As such, the bilingual researcher model is increasingly preferred as it acknowledges their active “physical and intellectual” participation in the research process as they interpret language and cultural information (Temple, 2002).

**References (Supplementary File 2)**

Björk Brämberg, E., & Dahlberg, K. (2013). Interpreters in cross-cultural interviews: A three-way coconstruction of data. *Qualitative Health Research, 23*(2), 241-247. <https://doi.org/10.1177/1049732312467705>

Liamputtong, P. (2008). *Doing cross-cultural research: Ethical and methodological perspectives*. Springer.

<https://search.ebscohost.com/login.aspx?direct=true&AuthType=sso&db=cat00006a&AN=melb.b3205873&site=eds-live&scope=site&custid=s2775460>

Temple, B. (2002). Crossed wires: Interpreters, translators, and bilingual workers in cross-language research. *Qualitative Health Research, 12*(6), 844-854. <https://doi.org/10.1177/104973230201200610>
