# Supplementary material for: Planning and conducting cross-cultural qualitative research: a methodological framework and resources for health researchers
Source: Int J Qual Stud Health Well-being. 2025 Oct 1;20(1):2556350. doi: 10.1080/17482631.2025.2556350 (PMC12490384; doi:10.1080/17482631.2025.2556350)
Supplement: SupplementaryFile1_Final.docx [file ZQHW_A_2556350_SM9317.docx]

**Supplementary File 1**

**Glossary of commonly used terminology in cross-cultural qualitative research**

| **Term** | **Definition** |
| --- | --- |
| Back translation | Reinstatement of a translated document to its original language usually by a third party (who was not involved in the initial translation) (Clark et al., 2017). |
| Bilingual researcher | Researcher actively engaged in the research process with the responsibility of also interpreting linguistic and cultural information from one language/culture to another (Temple, 2002). |
| Conceptual equivalence | Words, ideas, or concepts translated into another language so they convey similar meanings in each language after translation (Choi et al., 2012). |
| Cross-cultural | Cross-cultural refers to research intentionally conducted with participants from multiple cultural groups (Liamputtong, 2019). |
| Cultural broker | A cultural broker is someone in a position to enable clear communication of culturally specific needs to enhance understanding and engagement between individuals or groups and address cultural needs (McKenna et al, 2015). This term is more commonly associated with cultural brokerage between indigenous and non-indigenous groups in the literature. |
| Cultural insiders | Researchers may be considered cultural insiders when they share the language and culture of the study participants (Liamputtong, 2008). |
| Cultural integrity | Behaving in a manner consistent with participants’ cultural values (Pelzang & Hutchinson, 2017). |
| Cultural outsiders | Researchers may be considered cultural outsiders if they do not share the same culture and language of participants (Liamputtong, 2008). |
| Cultural reader | A research participant or someone with a similar cultural background to the research participants to read over the results or reports prior to dissemination to check they are reported in a culturally sensitive and safe manner (Burnette et al., 2014). |
| Cultural safety | The concept of cultural safety originated in the context of indigenous health care and has since evolved to include other cultural groups (Wilson & Neville, 2009). Cultural safety in research is about respecting the worldviews of research participants and recognising their cultural reality (which includes their historical, contemporary, socio-cultural and political realities) and considering these in the design of a study (Wilson & Neville, 2009). |
| Cultural sensitivity | Culturally sensitive research incorporates into its design and implementation  the historical context, and cultural experiences, norms, values, beliefs, and  behaviours of a distinct ethnic or cultural group (Burnette et al., 2014). |
| Culturally and linguistically diverse [CALD] | CALD refers to the range of different cultural and/or language groups represented in the population who identify as having particular cultural or linguistic affiliations due to their place of birth, ancestry or ethnic origin, religion, preferred language or language spoken at home (Woodland et al., 2021). This term is often used interchangeably with ‘migrant’ or ‘ethnic’. |
| Culture | Culture can be defined as the “shared beliefs, values and everyday practices groups of people undertake, influenced by their unique worldview” (Wilson, 2019). As this definition indicates – culture influences people’s perceptions of the world and their own life experiences (Wilson, 2019). Culture is defined and operationalised differently across disciplines (Kwantes & Glazer, 2017). For example, in anthropology the study of culture is considered through the cultural experiences of social groups (Kwantes & Glazer, 2017). Whereas in psychology, values and beliefs of individuals are studied to understand how individuals’ function within cultural systems or groups (Kwantes & Glazer, 2017). Culture is characterised as learned, not innate, and affects every aspect of human life (Kwantes & Glazer, 2017). Culture differs from race and ethnicity: ethnicity refers to ‘cultural groups’ of people based on heritage or nationality (Wilson, 2019). An individual’s culture is shaped by their social environment, including: family of origin, social contact with people from different cultural backgrounds to their own, and broader social and technological changes which influence social norms throughout one’s life course (Wilson, 2019). An individual’s awareness of their cultural milieu – where milieu refers to the range of factors which shape culture – may be implicit, explicit, or both (Kwantes & Glazer, 2017). As these contemporary definitions indicate, culture is complex, dynamic and evolving. |
| Intersectionality | The term intersectionality refers to the overlap - also referred to as ‘intersections’ - of multiple identities/characteristics/social categories such as gender and race (Kelly et al,. 2022) and that these identities are interrelated (Cole, 2009). Intersectionality acknowledges that membership within multiple social categories shapes individual meaning and experience as well as social representation (Carstensen-Egwuom, 2014). Intersectionality can be applied in research as a theoretical lens or methodology (combining epistemology and research methods) which underpins the research process (Carstensen-Egwuom, 2014). It can be used to examine the dimensions of power and oppression which may be shaping the experiences of a group with membership in multiple social categories as well as shared or differences in experiences among group members (Kelly et al., 2022). |
| Language assistant | The term language assistant represents either a language interpreter or bilingual researcher (Temple, 2002). |
| Language interpreter | Person responsible for interpreting information from one language to another. Usually, they have completing training and accreditation if employed formally in a professional role as a language interpreter (Temple, 2002). This model in research contexts is commonly viewed as having a singular role of interpreting language accurately and objectively. |
| Multicultural | The term multicultural describes a culturally diverse population or community (Woodland et al., 2021). |
| Translation | Translation of written documents from one language to another to convey the meaning of the original language in another language (Choi et al., 2012; Temple, 2002). Sometimes this term is used to represent both written and oral information being transferred from one language to another. |
| Verbal interpretation | Interpretation of verbal/oral information from one language to another (Temple, 2002). |

**References (Supplementary File 1)**

Burnette, C. E., Sanders, S., Butcher, H. K., & Rand, J. T. (2014). A toolkit for ethical and culturally sensitive research: An application with indigenous communities. *Ethics & Social Welfare, 8*(4), 364-382. <https://doi.org/10.1080/17496535.2014.885987>

Carstensen-Egwuom, I. (2014). Connecting intersectionality and reflexivity: Methodological approaches to social positionalities. *Erdkunde*, *68*(4), 265–276. http://www.jstor.org/stable/24365247

Choi, J., Kushner, K. E., Mill, J., & Lai, D. W. L. (2012). Understanding the language, the culture, and the experience: Translation in cross-cultural research. *International Journal of Qualitative Methods, 11*(5), 652-665. <https://doi.org/10.1177/160940691201100508>

Clark, L., Birkhead, A. S., Fernandez, C., & Egger, M. J. (2017). A transcription and translation protocol for sensitive cross-cultural team research. *Qualitative Health Research, 27*(12), 1751-1764. <https://doi.org/10.1177/1049732317726761>

Cole, E. R. (2009). Intersectionality and research in psychology. *American Psychologist*, *64*(3), 170. <https://doi.org/10.1037/a0014564>

Kelly, C., Dansereau, L., Sebring, J., Aubrecht, K., FitzGerald, M., Lee, Y., et al. (2022) Intersectionality, health equity, and EDI: What’s the difference for health researchers? *International Journal for Equity in Health,* 21(182). <https://doi.org/10.1186/s12939-022-01795-1>

Kwantes, C. T., & Glazer, S. (2017). *Culture, organizations, and work: Clarifying concepts*. Springer. <https://search.ebscohost.com/login.aspx?direct=true&AuthType=sso&db=cat00006a&AN=melb.b7186856&site=eds-live&scope=site&custid=s2775460>

Liamputtong, P. (2008). Chapter 1 Doing research in a cross-cultural context: Methodological and ethical challenges. In P. Liamputtong (Ed.) *Doing cross-cultural research: Ethical and methodological perspectives* (pp. 3-20). Springer. https://doi.org/10.1007/978-1-4020-8567-3_1

Liamputtong, P. (2019). *Handbook of research methods in health social sciences.* Springer. <https://search.ebscohost.com/login.aspx?direct=true&AuthType=sso&db=cat00006a&AN=melb.b7197133&site=eds-live&scope=site&custid=s2775460>

McKenna, B., Fernbacher, S., Furness, T. & Hannon, M*.* (2015) “Cultural brokerage” and beyond: Piloting the role of an urban Aboriginal Mental Health Liaison Officer. *BMC Public Health,* *15*, 881. <https://doi.org/10.1186/s12889-015-2221-4>

Pelzang, R., & Hutchinson, A. M. (2017). Establishing cultural integrity in qualitative research: Reflections from a cross-cultural study. *International Journal of Qualitative Methods, 17*(1). <https://doi.org/10.1177/1609406917749702>

Temple, B. (2002). Crossed wires: Interpreters, translators, and bilingual workers in cross-language research. *Qualitative Health Research, 12*(6), 844-854. <https://doi.org/10.1177/104973230201200610>

Wilson, D. (2019). Culturally safe research with vulnerable populations (Māori). In P. Liamputtong (Ed.), *Handbook of research methods in health social sciences.* Springer. <https://search.ebscohost.com/login.aspx?direct=true&AuthType=sso&db=cat00006a&AN=melb.b7197133&site=eds-live&scope=site&custid=s2775460>

Wilson, D., & Neville, S. (2009). Culturally safe research with vulnerable populations. *Contemporary Nurse, 33*(1), 69-79. <https://doi.org/10.5172/conu.33.1.69>

Woodland, L., Blignault, I., O’Callaghan, C., & Harris-Roxas, B. (2021). A framework for preferred practices in conducting culturally competent health research in a multicultural society. *Health Research Policy and Systems, 19*(1), 24. <https://doi.org/10.1186/s12961-020-00657-y>
